# Evaluation of droplet digital PCR for quantification of SARS-CoV-2 Virus in discharged COVID-19 patients

**DOI:** 10.18632/aging.104020

**Published:** 2020-11-01

**Authors:** Chong Liu, Qingxin Shi, Mingfei Peng, Ruyue Lu, Haohao Li, Yingying Cai, Jiaxi Chen, Jiaqin Xu, Bo Shen

**Affiliations:** 1 Zhejiang Taizhou Hospital Affiliated to Wenzhou Medical University, Taizhou 317000, China

**Keywords:** COVID-19, SARS-CoV-2, droplet digital PCR, discharged criteria

## Abstract

The worldwide severe acute respiratory syndrome coronavirus 2 (SARS-CoV-2) outbreak has led to the rapid spread of coronavirus disease (COVID-19). The quantitative real time PCR (qPCR) is widely used as the gold standard for clinical detection of SARS-CoV-2. However, more and more infected patients are relapsing after discharge, which suggests qPCR may fail to detect the virus in some cases. In this study, we selected 74 clinical samples from 43 recovering inpatients for qPCR and Droplet Digital PCR (ddPCR) synchronous blind detection, and established a cutoff value for ddPCR diagnosis of COVID-19. The results showed that at a cutoff value of 0.04 copies/μL, the ddPCR sensitivity and specificity are 97.6% and 100%, respectively. In addition, we also analyzed 18 retained samples from 9 discharged patients who relapsed. Although qPCR showed all 18 samples to be negative, ddPCR showed 12 to be positive, and there was only one patient with two negative samples; the other eight patients had at least one positive sample. These results indicate that ddPCR could significantly improve the accuracy of COVID-19 diagnosis, especially for discharged patients with a low viral load, and help to reduce misdiagnosis during recovery.

## INTRODUCTION

After the outbreak of the novel coronavirus disease (COVID-19), it quickly spread throughout China and to more than 180 other countries, and the World Health Organization (WHO) declared it to be a pandemic [1, 2]. Although quantitative real-time PCR (qPCR) detection of nucleic acids from the virus has become a standard method for diagnosis of SARS-CoV-2 infection, this approach is limited by an unsatisfactory amplification curve, which can lead to the occurrence of false negatives [3–5]. The high false negative rate and the lack of availability of qPCR assays for discharged patients during the outbreak restricted prompt diagnosis of infected patients [6, 7]. In addition, the qPCR method does not provide information about viral load, which would enable evaluation of disease progression and prognosis. Consequently, to prevent SARS-CoV-2 transmission and ensure timely treatment of infected patients, there is an urgent need for an accurate detection method to quickly identify infected patients and asymptomatic carriers [8].

Several studies have shown that droplet digital PCR (ddPCR) has the advantages of absolute quantification and greater sensitivity for virus detection than qPCR. In the present study, we used ddPCR to enable highly sensitive and quantitative detection of SARS-CoV-2 in discharged patients with low viral loads, which could prevent false negatives during clinical diagnosis, thereby reducing a potential risk of viral transmission. Through assays of 74 clinical samples, we were able to establish a SARS-CoV-2 cutoff value of ddPCR, which was then used to evaluate retained samples from discharged COVID-19 patients.

## RESULTS

### Comparison of ddPCR and qPCR

Seventy-four clinical samples collected from 43 recovering COVID-19 patients were tested using both qPCR and ddPCR. The samples including 36 feces samples, 36 sputum samples, and 2 throat swabs. Using qPCR, we found that 42 samples were positive and 32 were negative (Supplementary Table 1), and with ddPCR we found that 41 samples were positive and 33 samples were negative (Table 1). There were 16 samples with inconsistent results between the ddPCR and qPCR, which were considered positive, since all samples were from confirmed COVID-19 patients. ddPCR had a significantly higher positive detection rate than qPCR (55.41% vs. 36.49%; P<0.05, Chi square test).

**Table 1:** Comparison of ddPCR and qPCR in 74 clinical samples.

**qPCR**	**ddPCR**		**Total**
	**Positive**	**Negative**	
Positive	26	1	27
Negative	15	32	47
Total	41	33	74

### ddPCR sensitivity and specificity of SARS-CoV-2 assays in COVID-19 patients

**Figure 1:**
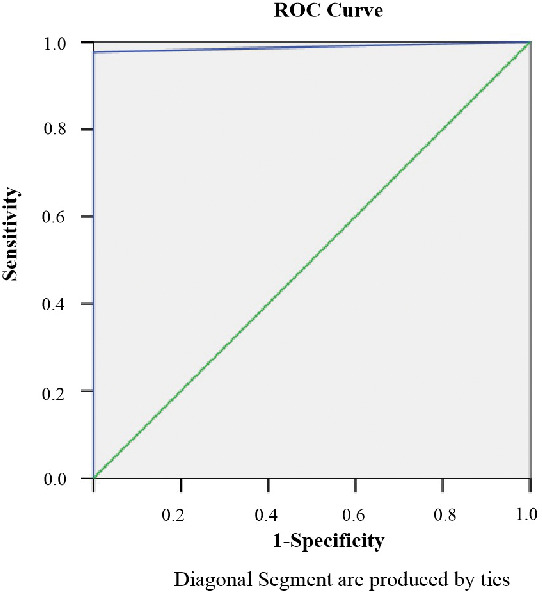
**The ROC curve for ddPCR.**

For 16 samples from COVID-19 patients, the results of SARS-CoV-2 virus detection with qPCR and ddPCR were not in agreement (Supplementary Table 1). After sample grouping, a receiver operating characteristic (ROC) curve was constructed for ddPCR (Figure 1). The area under the curve (AUC) was 0.988 (P<0.001), which indicates that the accuracy of ddPCR is very high. After selecting the point corresponding to the maximum value of the Youden index (Sensitivity value + Specificity -1) as the ddPCR cutoff value (0.04 copies/μL), the sensitivity was 97.6%, and the specificity was 100%, indicating moderate sensitivity and high specificity for SARS-CoV-2.

### Accuracy of ddPCR for discharge criteria for COVID-19 patients

The above results showed that ddPCR was better than qPCR for detecting SARS-CoV-2 in samples with a low viral load, especially in discharged patients. Therefore, We analyzed 18 retained samples from 9 discharged patients who had relapsed and were again testing positive. According to the qPCR results, all 18 retained samples were negative. However, ddPCR showed that 12 of the samples were positive, while only 6 were negative (Table 2). There was only one patient with two negative samples; the other eight patients had at least one positive sample (Table 2). This result indicates that ddPCR significantly improved diagnostic accuracy, especially for samples with a low viral load in supposedly recovered patients.

**Table 2:** Comparison of ddPCR and qPCR in 18 retained samples from 9 relapsed patients.

**Patient number**	**Sample and date twice**	**Result of official nucleic acid test by qPCR**	**qPCR (CtValue)**	**judgment result of ddPCR**	**Result of ddPCR copies/μl**
Patient1	Throat swabs (20200210)	Negative	NA	Positive	0.25
	Throat swabs (20200211)	Negative	NA	Positive	0.091
Patient2	Throat swabs (20200207)	Negative	NA	Positive	0.088
	Throat swabs (20200209)	Negative	NA	Positive	0.67
Patient3	Throat swabs (20200213)	Negative	NA	Positive	0.08
	Sputum (20200213)	Negative	NA	Positive	2.29
Patient4	Throat swabs (20200218)	Negative	NA	Positive	0.38
	Throat swabs (20200220)	Negative	NA	Negative	0
Patient5	Throat swabs (20200204)	Negative	NA	Negative	0
	Throat swabs (20200220)	Negative	NA	Negative	0
Patient6	Throat swabs (20200207)	Negative	NA	Negative	0
	Throat swabs (20200208)	Negative	NA	Positive	0.19
Patient7	Throat swabs (20200205)	Negative	NA	Positive	0.19
	Throat swabs (20200206)	Negative	NA	Positive	0.09
Patient8	Sputum (20200214)	Negative	NA	Positive	0.4
	Feces (20200216)	Negative	NA	Negative	0
Patient9	Throat swabs (20200221)	Negative	NA	Positive	0.086
	Feces (20200221)	Negative	NA	Negative	0

## DISCUSSION

The current method used for detection of SARS-CoV-2 involves a qPCR-based technique, which identifies the viral RNA when present in sufficient quantity [9]. Unfortunately, false negatives can occur, and the resultant failure to quarantine those infected patient would be a major setback to containing viral transmission [10]. When we compared the performances of qPCR and ddPCR in 74 samples from 43 confirmed patients, we found that qPCR and ddPCR gave comparable results with samples containing high viral loads, but ddPCR performed significantly better with samples containing a low viral load. Notably, the background readouts with ddPCR were lower, which could effectively reduce the incidence of false positives. Consequently, the ddPCR technique would likely be a better method for quantification of SARS-CoV-2 virus in discharged patients with a low viral load, since it shows superior precision and sensitivity for detection of low concentrations of target RNA [11, 12]. Yu et al. have taken a first step toward use of ddPCR for quantitative detection and viral load analysis of SARS-CoV-2 in infected patients [13]. Using ddPCR in the present study, we obtained 41 positives among our of 74 samples, and ddPCR showed outstanding sensitivity and specificity for SARS-CoV-2. These results thus suggest that for patients with a low viral load, SARS-CoV-2 virus amplification with ddPCR is technically feasible and could potentially be used as a standard method for dynamic detection of viral load.

More and more patients are showing relapse positivity after discharge, while numerous individuals test negative for COVID-19 many times before they test positive. This suggests that qPCR is failing to detect virus in some cases [14, 15]. According to the COVID-19 diagnosis and treatment plan used in China, patients reach the discharge criterion with at least two negative RNA tests. However, when we tested 18 retained samples from 9 discharged patients who were again testing positive, we found that only one patient with two negative samples; the other eight patients had least one positive sample. As time goes on, greater numbers of patients will enter the recovery stage and will be deemed ready for discharge. As a result, testing samples with low viral loads will become more frequent. We suggest that ddPCR should be used as a reference standard for discharge diagnosis of patients recovering from COVID-19. Moreover, ddPCR maybe used to monitor changes in viral load and to check the close contacts of patients who may be low virus carriers.

Due to its small sample size, our study has two limitations. First, we did not analyze the relationship between viral load and COVID-19 severity at different stages. Second, there was little analysis of the low viral load in discharged patients. In the future, we will investigate whether ddPCR can be used to dynamically monitor SARS-CoV-2 viral load in patients.

## MATERIALS AND METHODS

### Patients and samples

**Figure 2:**
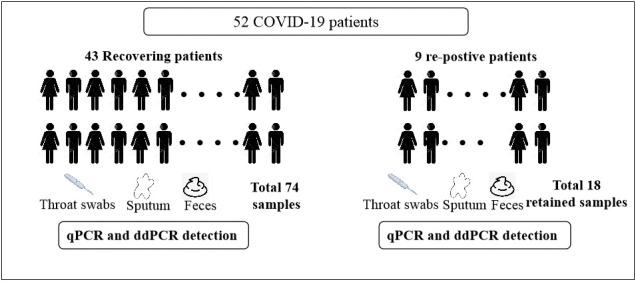
**Flow diagram of the study population.**

As shown in Figure 2, between February 29 and March 6, 2020, we collected 74 clinical samples from 43 recovering COVID-19 patients. These included 36 feces samples, 36 sputum samples, and 2 throat swabs. In addition, we selected 9 patients, who relapsed and were again positive for viral RNA after discharge. The 18 retained samples collected from these 9 patients before their first discharge included 3 sputum samples and 15 throat swabs.

All 52 patients gave informed consent and agreed to participate in this study. The patients included 27 (51.9%) males and 25 (48.1%) females with a median age of 51 years (range: 41 to 56 years). Among the participants, hypertension was found in 3 patients (5.8%), diabetes in 2 patients (3.8%), hyperlipidemia in 1 patient (1.9%), digestive diseases in 3 patients (5.8%), chronic liver disease in 4 patients (7.7%), chronic lung disease in 1 patient (1.9%), and a tumor in 2 patients (3.8%). In addition, 5 patients had undergone surgery (9.6%).

### Sample pretreatment and RNA extraction

Sputum was fluidized with a digestant, then centrifuged for 15 min at 14,000 rpm. Stool samples were homogenized in physiological saline, vortexed for 15 min, and centrifuged for 15 min at 10,000 rpm. Throat swabs were soaked in physiological saline and centrifuged for 15 min at 10,000 rpm. Following centrifugation, total RNA was extracted from the supernatants with a Nucleic Acid Extraction Kit, and processed according to the manufacturer's instructions (Shanghai ZJ Bio-Tech Co., Ltd).

### qPCR virus detection

qPCR detection was carried out by using a Novel Coronavirus Real Time qPCR Kit, and processed according to the manufacturer's instructions (Shanghai ZJ Bio-Tech Co., Ltd).

### ddPCR virus detection; primers and probe design

Primers and probes targeting the N gene of SARS-CoV-2 were designed by Primer Express software, while internal control (IC) gene was used to ensure the reagents work well. The sequences of primers and probes are shown in Table 3.

**Table 3:** The primers and probes for SARS-CoV-2 N gene and internal control.

	**Target N gene (5'-3')**	**Internal control (5'-3')**
Forward primer	CAACTCCAGGCAGCAGTAGGG	GGGCTCTTTGCAGGTCTCTC
Reverse primer	CTCTCAAGCTGGTTCAATCTGTCA	CCAGCAAGAGTCCCCATCC
Probe	CY5-AAGAGCAGCATCACCG-MGB	VIC-AGCCCCTTGTGGACATAGGGGTTT-BHQ1

### One-step RT ddPCR reaction

ddPCR was performed using a Droplet Digital PCR System (Pilot Gene Technologies (Hangzhou) Co., Ltd) according to the manufacturer’s instructions. Each one-step RT ddPCR reaction mixture contained 1x RT mix (Pilot Gene Technologies (Hangzhou) Co., Ltd), primers (final 1000 nM each), probes (final 250 nM each), and 5 μL of template in a final volume of 15 μL. A 14-μL aliquot of each reaction mixture was transferred for droplet generation, after which ddPCR and fluorescence reading was carried on a microfluidic chip. The thermal cycling protocol entailed incubation at 50°C for 30 min and 95°C for 10 min, followed by 45 cycles of 95°C for 15 s (denaturation) and 58°C for 1 min (annealing). The cycled chip was then transferred and read in the VIC, ROX and CY5 channels. A synthetic DNA fragment from the N gene served as a positive control, ultrapure water served as a negative template control, and a synthetic DNA fragment from the IC gene served as an internal control.

### Confirmation of the ddPCR cutoff

The 74 clinical samples were assayed using qPCR and ddPCR synchronously. After comparing the qualitative results of ddPCR and qPCR, samples for which the results differed between the two methods were classified as positive, since all samples were from confirmed COVID-19 patients. Other samples were classified based on consistent ddPCR and qPCR results. After sample grouping, a cutoff value for the ddPCR was confirmed using ROC curve analysis.

### Accuracy of ddPCR for discharged criteria for COVID-19 patients

Eighteen retained samples collected from 9 relapsed COVID-19 patients before they were first discharged were assayed using ddPCR and analyzed based on the ddPCR cutoff. The investigators performing the assays were blinded to the patient information.

### Statistical analysis

All statistical analyses were performed using SPSS 13.0 software.

### Ethical approval

The protocol was approved by the Ethics Committee of the Zhejiang Taizhou hospital affiliated to Wenzhou Medical University. Written informed consent was obtained from all participants.

## Supplementary Material

Supplementary Table 1
