# Supplementary material for: Evaluation of droplet digital PCR for quantification of SARS-CoV-2 Virus in discharged COVID-19 patients
Source: Aging (Albany NY). 2020 Nov 1;12(21):20997–1003. doi: 10.18632/aging.104020 (PMC7695381; doi:10.18632/aging.104020)
Supplement: Supplementary Table 1 [file aging-12-104020-s001..doc]

**Supplementary Table 1. Detection of ddPCR in 74 clinical samples from recovering COVID-19 patients.**

| Patient Number | Sample Number | Sample Type | Judgment result of qPCR | Result of ddPCR(copies/μL) | Judgment result of ddPCR | ddPCR vs. qPCR |
| --- | --- | --- | --- | --- | --- | --- |
| Patient01 | 202003017504 | Sputum | Negative | 0 | Negative | Identical |
| Patient02 | 202003017514 | Feces | Negative | 0 | Negative | Identical |
|  | 202003017519 | Sputum | Negative | 0 | Negative | Identical |
| Patient03 | 202003027517 | Feces | Negative | 0 | Negative | Identical |
| Patient04 | 202003017516 | Sputum | Negative | 0 | Negative | Identical |
| Patient05 | 202003017509 | Sputum | Negative | 0 | Negative | Identical |
| Patient06 | 202003017517 | Sputum | Negative | 0 | Negative | Identical |
| Patient07 | 202002297308 | Feces | Positive | 0.37 | Positive | Identical |
|  | 202002297310 | Sputum | Positive | 5.47 | Positive | Identical |
|  | 202003017310 | Sputum | Positive | 320.102 | Positive | Identical |
|  | 202003047546 | Sputum | Positive | 12.38 | Positive | Identical |
|  | 202003047550 | Feces | Negative | 0 | Negative | Identical |
|  | 202003067510 | Sputum | Positive | 2.1 | Positive | Identical |
| Patient08 | 202003047551 | Feces | Negative | 0.31 | Positive | Disaccord |
| Patient09 | 202002297322 | Feces | Positive | 9.2 | Positive | Identical |
| Patient10 | 202003067008 | Feces | Negative | 0 | Negative | Identical |
| Patient11 | 202003027501 | Throat Swabs | Negative | 0 | Negative | Identical |
| Patient12 | 202002297390 | Sputum | Negative | 0 | Negative | Identical |
|  | 202003027506 | Sputum | Negative | 0 | Negative | Identical |
| Patient13 | 202002297377 | Sputum | Negative | 0 | Negative | Identical |
| Patient14 | 202002297303 | Feces | Positive | 1.46 | Positive | Identical |
|  | 202002297313 | Sputum | Negative | 0 | Negative | Identical |
|  | 202003017303 | Feces | Positive | 0 | Negative | Disaccord |
|  | 202003017313 | Sputum | Negative | 0.262 | Positive | Disaccord |
| Patient15 | 202003017511 | Feces | Positive | 0.616 | Positive | Identical |
| Patient16 | 202003017392 | Sputum | Positive | 0.282 | Positive | Identical |
| Patient17 | 202002297307 | Feces | Negative | 0.22 | Positive | Disaccord |
|  | 202003017309 | Sputum | Negative | 0 | Negative | Identical |
|  | 202003027514 | Feces | Negative | 0.41 | Positive | Disaccord |
| Patient18 | 202003017512 | Feces | Negative | 0 | Negative | Identical |
| Patient19 | 202002297306 | Feces | Negative | 0.11 | Positive | Disaccord |
|  | 202003017306 | Feces | Negative | 0.218 | Positive | Disaccord |
|  | 202003027509 | Sputum | Negative | 0 | Negative | Identical |
| Patient20 | 202003057519 | Sputum | Negative | 0 | Negative | Identical |
| Patient21 | 202003067503 | Sputum | Positive | 0.11 | Positive | Identical |
| Patient22 | 202003027503 | Sputum | Positive | 3.29 | Positive | Identical |
|  | 202003027518 | Feces | Positive | 0.2 | Positive | Identical |
| Patient23 | 202003027502 | Sputum | Negative | 0 | Negative | Identical |
|  | 202003027513 | Feces | Negative | 0.32 | Positive | Disaccord |
| Patient24 | 202003027511 | Sputum | Negative | 0 | Negative | Identical |
|  | 202003027512 | Feces | Negative | 0.11 | Positive | Disaccord |
| Patient25 | 202003027508 | Sputum | Positive | 0.1 | Positive | Identical |
|  | 202003057516 | Feces | Negative | 0.18 | Positive | Disaccord |
|  | 202003057520 | Sputum | Positive | 1 | Positive | Identical |
| Patient26 | 202002297371 | Feces | Positive | 7.53 | Positive | Identical |
|  | 202003017312 | Sputum | Positive | 0.226 | Positive | Identical |
|  | 202003017393 | Sputum | Positive | 1.611 | Positive | Identical |
|  | 202003057517 | Feces | Positive | 15.14 | Positive | Identical |
| Patient27 | 202003027519 | Feces | Negative | 0 | Negative | Identical |
|  | 202003067011 | Feces | Negative | 0.081 | Positive | Disaccord |
| Patient28 | 202002297311 | Sputum | Positive | 4.44 | Positive | Identical |
|  | 202003017311 | Sputum | Positive | 0.384 | Positive | Identical |
| Patient29 | 202002297301 | Throat Swabs | Negative | 0 | Negative | Identical |
| Patient30 | 202003027520 | Feces | Positive | 11.31 | Positive | Identical |
| Patient31 | 202003057522 | Sputum | Positive | 1.11 | Positive | Identical |
| Patient32 | 202003027516 | Feces | Negative | 0 | Negative | Identical |
| Patient33 | 202003017506 | Sputum | Negative | 0 | Negative | Identical |
| Patient34 | 202002297302 | Feces | Negative | 0 | Negative | Identical |
|  | 202003017302 | Feces | Negative | 0.129 | Positive | Disaccord |
| Patient35 | 202002297305 | Feces | Negative | 0.15 | Positive | Disaccord |
|  | 202002297315 | Sputum | Negative | 0 | Negative | Identical |
|  | 202003027504 | Sputum | Negative | 0.55 | Positive | Disaccord |
| Patient36 | 202003027521 | Feces | Negative | 0 | Negative | Identical |
| Patient37 | 202002297379 | Sputum | Negative | 0.15 | Positive | Disaccord |
|  | 202003017513 | Feces | Negative | 0 | Negative | Identical |
|  | 202003057518 | Feces | Negative | 0 | Negative | Identical |
| Patient38 | 202003057512 | Feces | Negative | 0 | Negative | Identical |
|  | 202003057523 | Sputum | Negative | 0 | Negative | Identical |
| Patient39 | 202002297304 | Feces | Negative | 0 | Negative | Identical |
| Patient40 | 202003017391 | Sputum | Negative | 0.229 | Positive | Disaccord |
| Patient41 | 202003027522 | Feces | Positive | 5.43 | Positive | Identical |
| Patient42 | 202003017510 | Feces | Positive | 142.625 | Positive | Identical |
| Patient43 | 202003057515 | Feces | Positive | 2.57 | Positive | Identical |
|  | 202003067524 | Sputum | Positive | 23.97 | Positive | Identical |
